# Effectiveness of a digital alcohol moderation intervention as an add-on to depression treatment for young adults: study protocol of a multicentre pragmatic randomized controlled trial

**DOI:** 10.1186/s12888-021-03222-1

**Published:** 2021-05-22

**Authors:** Maria J. E. Schouten, Jack J. M. Dekker, Tamara Q. de Bruijn, David D. Ebert, Lisanne M. Koomen, Sjoerd L. A. Kosterman, Heleen Riper, Michael P. Schaub, Anna E. Goudriaan, Matthijs Blankers

**Affiliations:** 1 Department of Research, Arkin Mental Health Care, Amsterdam, The Netherlands; 2 Department of Clinical, Neuro- and Developmental Psychology, Vrije Universiteit Amsterdam, Amsterdam Public Health research institute, Amsterdam, The Netherlands; 3 Department of Prevention, Jellinek, Arkin Mental Health Care, Amsterdam, The Netherlands; 4 Department of Clinical, Neuro- and Developmental Psychology, Vrije Universiteit Amsterdam, Amsterdam, the Netherlands; 5 Arkin BasisGGZ, Arkin Mental Health Care, Roetersstraat 210, Amsterdam, The Netherlands; 6 Department of Outpatient Treatment of Common Mental Health Disorders, PuntP, Arkin Mental Health Care, Amsterdam, The Netherlands; 7 Department of Research and Innovation, GGZ inGeest Specialized Mental Health Care, Amsterdam, The Netherlands; 8 Amsterdam UMC, Vrije Universiteit Amsterdam, Psychiatry, Amsterdam Public Health Research Institute, Amsterdam, The Netherlands; 9 Research Unit for Telepsychiatry and e-Mental Health, Department of Clinical Research, University of Southern Denmark, Odense, Denmark; 10 Faculty of Medicine, University of Turku, Turku, Finland; 11 Swiss Research Institute for Public Health and Addiction, University of Zurich, Zurich, Switzerland; 12 Department of Psychiatry, Amsterdam UMC, University of Amsterdam, Amsterdam Institute for Addiction Research and Amsterdam Public Health Research Institute, Amsterdam, The Netherlands; 13 Trimbos Institute–The Netherlands Institute of Mental Health and Addiction, Utrecht, The Netherlands

**Keywords:** Randomized controlled trial, Depressive disorders, Problematic alcohol use, Digital intervention, Comorbidity, Young adults, Depression treatment, Effectiveness, Cost-effectiveness, Ehealth

## Abstract

**Background:**

Depressive disorders and problematic drinking often co-occur, also among young adults. These co-occurring conditions are associated with various negative health outcomes compared to both conditions alone. Early intervention by addressing alcohol use and depressive symptoms simultaneously in the same treatment might improve both conditions. However, evidence on the (cost-) effectiveness of digital combined depression and alcohol interventions for young adults is currently insufficient. We therefore developed an add-on digital alcohol moderation adherence-focussed guided intervention to complement treatment as usual (TAU) for depressive disorders. The digital intervention is a web-app, including 6 modules based on motivational interviewing and cognitive behavioural therapy. This study aims to evaluate the (cost-)effectiveness of a digital alcohol moderation intervention + TAU compared to TAU on alcohol and depression outcomes among young adults with co-occurring depressive disorders and problematic alcohol use.

**Methods:**

One hundred fifty-six participants, aged 18–35 years, with problematic alcohol use and a diagnosed depressive disorder will participate in a pragmatic multicentre two-arm randomized controlled trial. Problematic alcohol use is operationalised as scoring ≥5 for women and ≥ 8 for men on the Alcohol Use Disorder Identification Test (AUDIT). Participants will be randomized to either the experimental group (digital alcohol intervention + TAU) or control group (TAU only). Participants will be recruited at three Dutch mental health care centres and through social media. Assessments take place at baseline and after 3, 6 and 12 months post-randomization. The primary outcome is treatment response at 6-month follow-up, operationalized as a composite score that combines alcohol use and depression measures and indicates whether treatment has been successful or not. Secondary outcomes are depressive symptoms and alcohol use (i.e. number of weekly standard drinks and AUDIT score). An economic evaluation will be conducted alongside the trial.

**Discussion:**

This study evaluates the (cost-) effectiveness of an add-on digital alcohol moderation intervention for young adults who are in treatment for depressive disorders. If proven effective, the digital intervention could be implemented in mental health care and improve treatment for people with co-occurring depressive disorders and problematic alcohol use.

**Trial registration:**

Pre-registered on October 29, 2019 in The Netherlands Trial Register (NL8122).

## Background

Depressive disorders are common among adolescents, young adults and students, with lifetime prevalence rates up to 11–15, 27 and 21%, respectively [1–4]. Its onset is often in young adulthood and is associated with various adverse outcomes in mental health, education and employment in later adult life [1, 2, 5–8]. Alcohol use disorders (AUD) and other forms of problematic alcohol use, such as non-clinical levels of hazardous drinking, often co-occur with depressive disorders [9, 10]. Among young adults, lifetime prevalence of co-occurring depressive disorders and problem drinking is estimated to be up to 20% [11]. Co-occurring depressive disorders and problematic alcohol use are associated with higher risk of developing severe AUD, suicide attempts and greater disease burden and with lower life satisfaction and functioning compared to both conditions alone [11–13]. Considering these negative consequences in both current and later adult life, it is of great importance to intervene early among young adults who experience both concurrent conditions. By addressing alcohol use and depressive symptoms simultaneously in the same treatment setting, improvements may be reached in both.

Psychological treatment for co-occurring depressive disorders and problematic alcohol use often includes elements of motivational interviewing (MI) and cognitive behavioural therapy (CBT). Both treatment techniques have been found to be effective in reducing either problematic alcohol use [14–16] or depressive symptoms [17] among young people. In addition, two literature reviews have found MI/CBT-based treatments, often face-to-face delivered, to be effective in reducing both alcohol use and depressive symptoms simultaneously among people with co-occurring depression and problematic alcohol use [18, 19]. However, implementation of face-to-face delivered psychological treatment is often challenged by high costs and limitations in scalability. Contrary, digital-delivered treatment might overcome some of these challenges and therefore increase access to evidence-based psychological treatments [20, 21]. This is of great importance, especially considering the expected increase in demand for mental health care in the forthcoming years and limited healthcare resources [22–24]. In addition, important barriers for students to not seek help in case of future emotional problems appear to be stigma and preferring to solve problems alone. Hence, digital-delivered self-help interventions might especially be suitable for this particular young population that is reluctant to seek help [25].

Digital self-help interventions are delivered by internet, mobile device or computer and exist in guided and unguided forms. Digital interventions are not only promising due to young peoples’ increased usage of internet and mobile devices, but also because other potential benefits such as accessibility, reach, perceived anonymity and blended treatment where digital interventions are integrated into traditional treatment settings [24, 26–33]. Furthermore, digital alcohol moderation interventions might target people who are less likely to access traditional substance use facilities, such as women, young people and people who drink alcohol at problematic levels [34]. Young people also appear to already regularly use digital mental health services and their experiences with it are generally positive [35]. Notably, students seem to prefer digital treatment over face-to-face treatment for stigmatizing problems and anonymous alcohol interventions over formal alcohol resources such as talking to a doctor [36, 37]. Accordingly, an add-on digital alcohol moderation intervention to regular depression treatment may be an acceptable intervention for young adults with co-occurring depressive disorders and problematic alcohol use.

To date only a few randomized controlled trials (RCTs) have been conducted on evaluating digital interventions for co-occurring depressive disorders and problematic alcohol use. Contrary, various systematic reviews and meta-analyses have already shown that digital interventions, though mostly focused on only one condition, are effective in reducing depressive symptoms in youth [27, 38], adolescents [27], students [39, 40] and in adults [41, 42] and in reducing alcohol consumption among young adults [43], students [44] and adults [45–47]. A few meta-analytic studies have been conducted on digital interventions for co-occurring depressive disorders and problematic alcohol use. A sub-group meta-analysis from 2014, restricted to exclusively MI/CBT-based interventions, showed digital MI/CBT interventions may even reduce depression outcomes to a larger extent than face-to-face MI/CBT treatment in adults with co-occurring depression and problematic alcohol use [18]. Our recent systematic review with meta-analysis on digital interventions for co-occurring depression and problematic alcohol use also provides preliminary evidence of the small but significant positive effects of digital interventions on reducing depressive symptoms after 3-month follow-up (g = 0.34) and alcohol use after 6-month follow-up (g = 0.14) [48]. Digital interventions seem therefore particularly promising for this comorbid population. However, these meta-analytic findings are limited by high risk of bias ratings for all included studies, small amount of included studies with sometimes small sample sizes and differences with regards to digital intervention and population characteristics [48]. Furthermore, only two trials were conducted among younger populations and report mixed findings [49, 50]. Geisner et al. [49] found no effects for a brief web-based personalized feedback intervention on depression and alcohol outcomes among students with co-occurring problematic alcohol use and depressed mood. Deady et al. [50] evaluated a four-hour module web-based self-help for co-occurring depression and problematic alcohol use in young people aged 18–25 years. The authors found statistical significant effects in favour of the digital intervention compared to the control condition in depression and alcohol use outcomes post-treatment, but these group differences were not maintained after 3- and 6-months follow-up. Furthermore, no economic evaluations of digital interventions for co-occurring depressive disorders and problematic alcohol use have been conducted.

The previously discussed literature indicates that a digital alcohol moderation intervention might be a suitable and effective treatment for reducing depressive symptoms and alcohol use simultaneously among young adults with these co-occurring conditions. However, evidence on (cost-)effectiveness of digital interventions for young adults with these co-occurring conditions is insufficient. Therefore, we developed “Beating the Booze” (BtB) an add-on digital alcohol moderation adherence focussed guidance intervention as an adjunct to treatment as usual (TAU) for depressive disorders for young adults with co-occurring depressive disorders and problematic alcohol use. In the RCT presented in this protocol paper, we aim to evaluate the (cost-)effectiveness of this add-on digital alcohol intervention.

## Methods

This study protocol describes a multicentre RCT in which the (cost-) effectiveness of combining an add-on digital alcohol moderation intervention (BtB) with TAU will be compared to TAU alone. The target population comprises young adults (aged 18–35 years) with co-occurring depressive disorders and problematic alcohol use. We will evaluate alcohol use and depression outcomes. This protocol was written in accordance with the Standard Protocol Items: Recommendations for Interventional Trials (SPIRIT) guideline [51].

### Research aims

The primary outcome of the RCT is treatment response after 6-months follow-up. Treatment response is defined as a composite score that combines alcohol and depression outcome measures and indicates whether treatment has been successful or not. We expect to find a 25% treatment response in the control condition (TAU) and a 50% treatment response for the experimental condition (TAU + BtB). Previously conducted and ongoing trials indicate that changes in order of this magnitude can be achieved for combined digital alcohol and depression interventions [52, 53]. Furthermore, compared to participants in the control condition, we expect participants in the experimental condition to report a larger decrease in outcomes measuring alcohol use and depressive symptoms and a larger increase in quality of life.

### Design

**Fig. 1:**
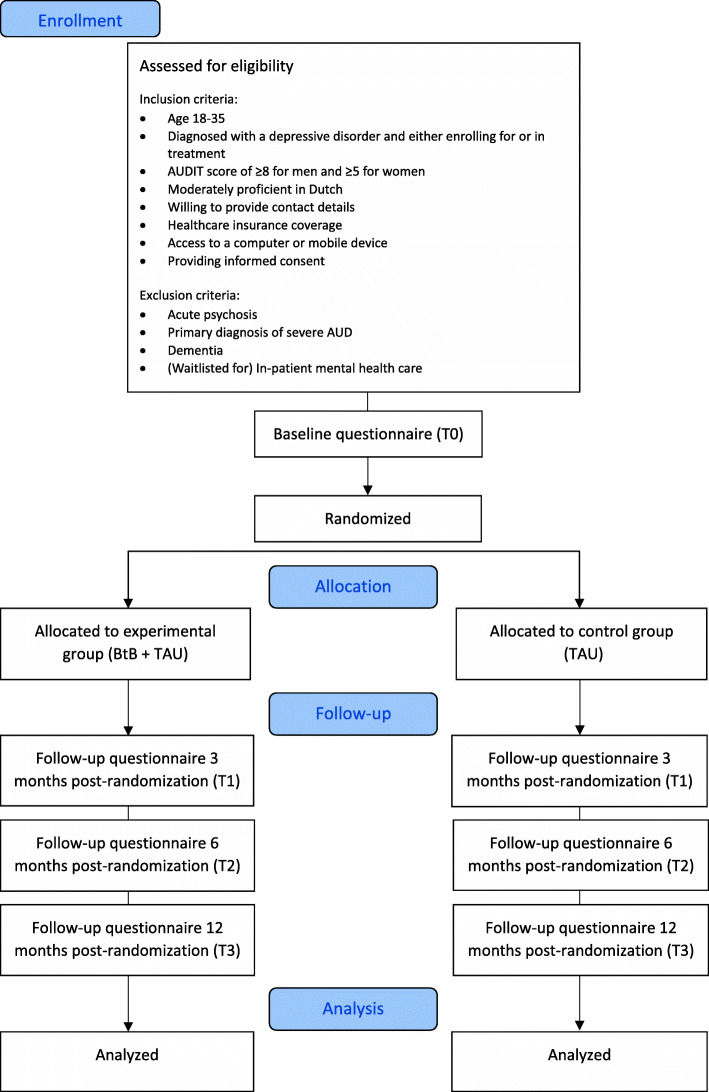
Flowchart of study inclusion

We will conduct a pragmatic two-arm single-blind multicentre RCT with parallel-group design. Follow-up assessments will take place after 3 (T1), 6 (T2 and primary endpoint RCT) and 12 (T3) months post-randomization, see Fig. 1 for a flowchart of the study. The baseline and follow-up assessments are self-report measures and will be collected as online questionnaires. Where possible, we will use patient records to complement the self-reported data.

### Economic evaluation

We will perform an economic evaluation alongside the RCT following a piggyback approach, to evaluate the cost-effectiveness of the intervention compared to care as usual. The economic evaluation will be performed in line with the International Society for Pharmacoeconomics and Outcomes Research (ISPOR) guidelines [54].

### Ethical approval

The research protocol was submitted to and approved by the Medical Research Ethics Committees United (MEC-U) in the Netherlands (NL66899.100.18). The research will be conducted in accordance with the Helsinki declaration and is pre-registered at the Netherlands Trial Register under registration number NL8122 (https://www.trialregister.nl/trial/8122) [55]. All adverse events reported spontaneously by the subject or observed by a member of the research team will be recorded. Any serious adverse events will be reported to the MEC-U. Written informed consent is obtained from all participants included in the study.

### Participants

The target population consists of Dutch young adults with a depressive disorder, 18 to 35 years old, who drink alcohol at least at hazardous levels and receive outpatient treatment for a depressive disorder. Participants will be recruited at three participating sites of Arkin Mental Health Care (the Netherlands) and through social media advertisements. Patients are eligible to participate in the study if they meet the following inclusion criteria: (1) between 18 and 35 years old, (2) diagnosed with a depressive disorder and either enrolling for treatment or currently in treatment (3) a score of ≥8 for men and ≥ 5 for women on the Alcohol Use Disorders Identification Test (AUDIT), (4) at least moderately proficient in Dutch, (5) willingness to provide contact details, (6) healthcare insurance coverage, (7) access to a computer or mobile device and (8) providing informed consent. Potential participants are excluded from study participation in case of: (1) acute psychosis, (2) having a primary diagnosis of severe AUD, (3) dementia, (4) (waitlisted for) in-patient mental health care or (5) pregnancy.

### Sample size

We used R package ‘pwr’ to calculate the sample size with α = .05 (two-sided) and Power (1-β) = .80 [56]. We aim to include a total of 156 participants to detect a 25%-point treatment effect difference between experimental and control groups (i.e. treatment response of 50 and 25%, respectively). Sample size is calculated by taking into account 35% extra inclusion to account for drop-out and the design effect (multicentre clustering).

### Procedures

#### Recruitment

Study participants will be recruited at three mental health care sites where treatment for depressive disorders is provided. These sites differ in their patient population. The first site focuses exclusively on youth (0–23 years), the second on adults who experience mild to moderate mental health problems and the latter is focused specifically on mood- and anxiety disorders. Applicants at one of these sites will be pre-screened for eligibility using the AUDIT-C included in the baseline measurement of the routine outcome monitoring assessment [57]. Potential participants who agree to be approached by a research team member are contacted and informed about the study and any questions from the applicants will be answered. Consequently, a detailed assessment of eligibility based on all inclusion and exclusion criteria will be conducted. Eligible participants receive detailed written information about the study and those who are willing to participate sign the informed consent form and receive an invitation for the T0 assessment no sooner than 4 weeks before the start of their treatment.

Regarding social media recruitment, young adults who recently started or soon will start depression treatment throughout the Netherlands are invited through Facebook and Facebook audience network advertisements (e.g. Instagram, Messenger) to fill out an online screening questionnaire to check for eligibility to participate in the study. A short summary of the study is provided prior to filling out the screening questionnaire. Eligible participants receive detailed written study information and contact details of the research team in case they have further questions. Next, they are invited to fill out the informed consent form. The informed consent form is validated in a personal contact between a member of the research team and the participant. After validated informed consent, the contact information of the participant’s therapist is obtained. The therapist is then informed about the study and the participant’s intention to participate in the study. If the therapist does not have any objections, the participant can participate in the study and the research member invites the participant to fill out the online T0 assessment.

#### Randomization and follow-up assessment

After completion of the baseline assessment, participants will be randomly allocated to the experimental or control group in a 1:1 ratio. Randomization will be performed using Castor EDC, a clinical data management system [58]. Using variable block randomization with block sizes of 2 and 4, randomization will be stratified for every participating site and for the group of participants recruited through social media. The randomization sequence is concealed for all members of the research team.

Castor EDC will be used for sending and storage of all online assessments [58]. All follow-up assessments will be online self-report questionnaires after 3, 6 (RCT primary endpoint) and 12 months post-randomization. Participants are e-mailed a link to the online questionnaires which they can fill-out on their mobile device (e.g. smartphone, computer or tablet). Participants who do not complete an assessment within 4 days will receive a reminder via e-mail. Participants who have not completed the assessments after the reminder, will be contacted by a research team member who motivates them and can assist them with possible assessment problems. If necessary, online assessments can also be completed by telephone. Research team members involved in follow-up assessments will be blinded to participants treatment allocation. Participants will receive a gift card with a value of €20 for each completed follow-up measurement assessment.

### Add-on digital alcohol moderation intervention: “Beating the Booze”

#### Intervention development

The development of the BtB (“Beating the Booze”) web-app for young adults with depressive disorders is based on an existing and implemented alcohol web-based self-help by Jellinek, a Dutch health care facility specialised in treatment of addiction, which has been found to be effective in reducing alcohol use among general population problem drinkers in previous research [59]. We adjusted this programme to fit the needs and preferences of our young target group with depressive disorders. BtB was developed through co-creation with experts by experience and by consultation with addiction and eHealth professionals. The experts by experience included young adults (18–35 years) who had experience with either depression treatment or problematic alcohol use or both. We organized two rounds of focus groups to gain insight in the preferences of the experts by experience, regarding the BtB intervention. The first round of focus groups was aimed at exploring needs and preferences on the intervention’s look and content of 15 experts by experience. We made a prototype of BtB based on the results from these focus groups. Then 14 experts by experience, including 10 new and 4 participants who also participated in the first round, provided direct feedback on the prototype in the second round of focus groups. Based on the input from this second round of focus groups and consultations with professionals, final adjustments to the intervention were made.

#### Intervention content

BtB is a web-app and includes 6 CBT/MI-based modules with psycho-education and assignments. BtB has a responsive web-design and is therefore made accessible for mobile devices such as computer, smartphone and tablet. The intervention addresses both alcohol use and depression and their underlying interaction, but has a main focus on reducing alcohol use. The modules are aimed at reducing alcohol use according to the participants’ personal drinking goal. Another core feature of the program is aimed at self-monitoring of alcohol consumption. Participants are encouraged to report their daily alcohol use in order to attain insight in drinking patterns and the progress in adhering to their personal drinking goal. Optional alcohol registration features include registering where, with whom and what the feelings and thoughts were when the participant drank alcohol. Data from these alcohol use registries are displayed in graphs to provide the participant visual insight in their drinking patterns.

#### Account registration and personal goal setting

Participants start account registration by filling out their e-mail address, name, anonymous nick-name and password. Consequently, they are routed to a short survey that is aimed at determining their personal drinking goal. Participants first fill out their alcohol consumption on a weekly calendar and then choose their drinking goal that they wish to achieve during the BtB program, that is either abstinence or controlled drinking. In case of controlled drinking, participants are asked to fill out on which days of the week they want to drink and the maximum amount of standard drinks. Controlled drinking goals have to be lower than the users’ current weekly alcohol intake. If preferred, participants can gradually reduce their current alcohol intake towards their drinking goal by following a reduction scheme. Participants can start the program by either personalizing their account, for example by choosing an animated avatar, personalize notifications settings and inviting a ‘buddy’ who receives e-mails about the participants’ program progress or they can immediately start with the first module.

#### CBT−/MI-based modules

BtB is designed as a modular self-help with 5 modules and 1 aftercare module, which participants can complete in their own pace. Each module contains psychoeducation on a specific theme and includes animated video (90 seconds), short written text (i.e. “reading assignments”), assignments, patient stories (either in form of short quotes added to reading assignments or elaborated stories as a reading assignment) and a short assessment in which the module’s key points are summarized. Each module can be completed in multiple sessions and takes about 30–45 min to be completed. The six modules have to be completed in ascending order. In consultation with eHealth professionals specialized in substance use self-help programs, we have set time locks of 5 and 3 days on certain modules. This helps to ensure that participants have enough time to apply the gained skills into practice and to register their alcohol intake to gain insights in to drinking patterns. Therefore, after completing the second module, participants have to register their alcohol intake for 5 days before the third module is unlocked. A time lock of 3 days is used for every following module, which will be activated after completing every module.

**Fig. 2:**
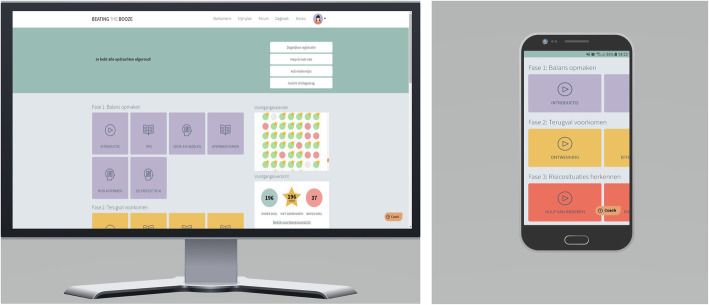
Beating the Booze dashboard: computer and smartphone

See Table 1 for a detailed overview of all the elements of the intervention and Fig. 2 for images of BtB. The content of every module is as follows:

- Module 1. Changing alcohol use: starts with an introduction video about BtB, followed by program-related tips (e.g. instructions for installing the web-app on a smartphone). In the first assignment the participant writes down the disadvantages of their current alcohol use and the advantages of changing their alcohol use. These personal advantages will be displayed on the participants’ program dashboard for motivation. The reading assignment includes information about self-control strategies that may help in adhering to their drinking goal, followed by an assignment in which the participant writes down his or her personal measures that they intend to use. Lastly, the participant fills out a short summary assessment in which the main points of module 1 are summarized.
- Module 2. Preventing relapse: starts with a video about how to deal with withdrawal symptoms. Reading assignments include information about physical and mental short- and long-term effects of alcohol, dealing with craving and learning from a relapse. In the assignments participants determine their high risk situations and make a plan about how to react in case of a relapse. An optional assignment consists of making a personal activity list, in which the participant “likes” or “dislikes” sets of activities. Activities that are rated with a “like” are followed by a second rating in which the participant answers if he or she usually drinks alcohol during this activity. The activities that are “liked” and do not include drinking alcohol are added to the personal activity list. Additional activities can also be added to the list by the participant. The module is completed after filling out the summary assessment of module 2.
- Module 3. Recognizing and dealing with high risk situations: starts with a video about receiving help from others, such as friends or family. Reading assignments include information about high risk situation that might lead to relapse and an alcohol and depression-related patient story. Assignments include identifying and writing down personal high risk situations, an optional quiz about alcohol facts and lastly a summary assessment about module 3.
- Module 4. Gaining insight: starts with a video about how to decline alcoholic drinks in social situations, followed by gaining insight in drinking patterns by studying registration graphs that show with whom, where and what feelings and thoughts the participant usually has when drinking. These insights may help with recognizing high risk situations. Other assignments include making a prevention plan in which the participant writes down how to react in case of high risk situations and filling out the summary assessment of module 4. If preferred, the participant can update his or her activity list.
- Module 5. Restructuring thoughts: starts with a video about helping thoughts and reading assignments including information about restructuring negative thoughts into positive thoughts and a patient story. Assignments are aimed at restructuring thoughts and optional assignments include a quiz about alcohol facts. Lastly, the module is completed after the summary assessment is finalized.
- Module 6. Staying motivated: The last module is an aftercare module and starts with a final video about the completion of the program and how to continue. Reading assignments include information about which program features remain accessible after program completion, referral to other forms of (intensive) treatment options and a patient story. The module is completed after a final summary assessment in which the participant reflects on personal achievements and challenges regarding their drinking goal during the program.

**Table 1:** Content and features of Beating the Booze

Phase	Element	Content
**Create & set up account**	Determining personal drinking goal	Abstinence: gradually reduce alcohol use until abstinence. Controlled drinking: set the maximum of drinks per day and number of drinking days.
	Setting up personal profile, preferences for program reminders	Choosing animated avatar, name, preferences for receiving program reminders (e.g. registering alcohol use, progress report after completing modules, notification for reply’s on forum boards). Optional: inviting a buddy who receives progress reports.
**Getting started**	Daily alcohol use registration during the program	Registration and monitoring of daily alcohol use and if preferred, registration of where and with whom and what type of thoughts and feelings the participant experienced. Optional: registration of mood and activities during the day.
	Module 1: Changing alcohol use	Animated video: introduction about the program. Reading assignment: tips for the optimal program experience. Assignment: writing down disadvantages of current alcohol use and advantages of changing alcohol use in short- and long-term. Reading assignment: information on self-control strategies that may help to adhere to the drinking goal. Assignment: writing down personal self-control strategies. Summary assessment: self-reflection questions in which the key points of module 1 are summarized.
	Module 2: Preventing relapse	Animated video: information on withdrawal symptoms. Reading assignment: physical and mental short- and long-term effects of alcohol. Reading assignment: dealing with craving. Optional assignment: setting up a personal activity list of things to do. Reading assignment: learning from a relapse. Assignment: writing down a personal relapse plan. Summary assessment: self-reflection questions in which the key points of module 2 are summarized.
	Module 3: Recognizing & dealing with high risk situations	Animated video: information about how help from others can help in adhering to drinking goal. Reading assignment: identifying high risk situations. Assignment: writing down personal high risk situations. Optional Quiz 1: facts about alcohol and depression. Reading assignment: patient story. Summary assessment: self-reflection questions in which the key points of module 3 are summarized.
	Module 4: Gaining insight	Animated video: skills to decline offered alcoholic drinks. Assignment: insight in to personal drinking patterns (graphs that visually display frequent locations and persons and experienced thoughts and feelings from when the participant drinks alcohol and that were registered during the program). Assignment: writing down a personal prevention plan. Optional assignment: update the personal activity list. Summary assessment: self-reflection questions in which the key points of module 4 are summarized.
	Module 5: Restructuring thoughts	Animated video: information about restructuring and challenging negative thoughts. Optional Quiz 2: facts about alcohol. Reading assignment: information about the relationship between thoughts, feeling and behaviour. Assignment: challenging negative thoughts by writing down helping thoughts. Reading assignment: patient story. Summary assessment: self-reflection questions in which the key points of module 5 are summarized.
	Aftercare module 6: Staying motivated	Animated video: final video that summarizes phase 1–5. Reading assignment: information about other treatment resources for reducing alcohol use. Reading assignment: patient story. Final summary assessment: self-reflection questions about achievements and challenges regarding changing alcohol use.
**Extra (optional) features**	Diary	Free writing in diary.
	Forum	Various forum boards about depression and alcohol use, to exchange positive personal stories and motivate and talk to other peers.
	Dealing with craving	Tips and assignments about how to cope with craving.
	Additional information	Additional information and tips for: alcohol-free drinks, tips to stay motivated, how to deal with stress, tips to sleep better, definition standard glasses, dealing with negative feelings, mindfulness exercises.
	Insight in alcohol use patterns and high risk situations	Visual graphs: displays with whom and which locations and specific thoughts and feelings that the participant registered and experienced when drinking alcohol.
	Progress report	Monthly calendar: displays the registered days in which participant did or did not adhere to their drinking goal. Days that light up green indicate drinking below drinking goal and red indicate drinking above drinking goal.
	Milestones & badges	Earning badges for program activities and achievements: e.g. adhering to drinking goal for certain amount of time, writing in diary, posting messages on the forum boards, completing modules.
	Program progress reports e-mails	Personalized progress reports of completed modules.

#### Optional program elements

Other optional features of BtB include forum boards, additional information, diary, progress reports on alcohol use patterns and drinking goal achievement and registering of daily mood and activities. Participants will receive automatic e-mails with reminders to log in after periods of inactivity and to inform if new modules are accessible.

#### Duration

Previous research implies that participants do not always have to complete all of the available elements of a digital intervention to achieve beneficial effects. Participants also might have different effective usage patterns and thus might use different intervention elements compared to other participants depending on their personal goal [60]. The duration of BtB will vary for every participant since the program can be completed in the participants own pace. At fastest, that is the minimal duration of completion of all modules, is 18 days. We however advise participants to complete every module within 1 or 2 weeks. The combined treatment, including TAU + BtB has a per-protocol duration of 4–6 months.

#### Guidance

BtB includes a minimal level of asynchronously delivered adherence-focussed (i.e. not care-related) guidance from a “coach”. Guidance is performed by a member of the research team. Participants can also initiate contact with the coach by sending a message within the self-help environment. Guidance will be facilitative in nature, that means that it will be focused on increasing program adherence by answering any (technical) program related questions and motivating and reminding people to log in after a certain time of inactivity. The guidance will be tailored to the activity level of the participant, meaning that participants with low level of activity in the program will receive more reminders than participants who frequently log in to the program.

### Treatment as usual

Both experimental and control condition will receive TAU, that is regular treatment for depressive disorders provided as routine care. The TAU often has a duration of 4–6 months and often consists of 8–16 sessions of regular CBT or other evidence-based psychotherapy (e.g. interpersonal psychotherapy, problem solving therapy), if necessary combined with medication. TAU is often aimed at activation, identification and restructuring of maladaptive cognitions and is offered at the participating sites [61]. Commonly, each session follows a fixed format, including agenda setting, explanation of rationale of each session, review of homework assignments and assigning new homework. The TAU can be either 100% face-to-face delivered with paper-based assignments, blended, that is partially or fully face-to-face delivered therapy with internet-based homework assignments and/or contacts, or 100% digitally (screen-to-screen) delivered therapy.

### Outcome measures

#### Primary outcome measure

The primary outcome is treatment response (yes/no) and is measured at 6-month follow-up which is the primary endpoint of the RCT. The treatment response outcome combines an alcohol use measure and a depression-related measure into a composite score. Alcohol use will be measured with the self-administered 7-day Timeline Followback (TLFB) methodology [62]. The TLFB is a frequently and widely used retrospective calendar method of assessing daily alcohol use estimates in a certain period of time (e.g. 7 or 30 days). The self-administered web-based 30-day TLFB on drinking has shown strong psychometric properties among young adults and research suggests that the 7-day TLFB may be more accurate in assessing volume and timing of consumption compared to the 30-day TLFB due to longer-recall periods [63, 64]. In addition, there is evidence supporting that individuals may feel more comfortable completing the TLFB online [65]. Depression is measured with the brief 20-item self-report Center for Epidemiological Studies-Depression (CES-D) and measures current depression symptoms a week prior to assessment. CES-D scores range between 0 (best possible) and 60 (worst), a cut-off point of 16 is often recommended for detecting depression [66]. The CES-D has acceptable screening accuracy in both general population and primary care settings [67]. Treatment is deemed successful (i.e. treatment response) if all following three conditions are met: (1) drinking less than 21 (males) or 14 (females) glasses of alcohol in the week prior to measurement, (2) 0 days with 4 or more (women), or 5 or more (men) drinks reported in the last 7 days and (3) a CES-D depression score of < 16 or a reduction of 40% relative to CES-D at baseline. Glasses are in this study defined as standard drinks which contain 10 g of ethanol (the European standard).

#### Secondary outcome measures

Secondary outcome variables in this study are: alcohol use (TLFB), depressive symptoms (CES-D) and quality of life. Alcohol use is measured in addition to the 7-day TLFB with the AUDIT [62, 68]. The AUDIT is a 10-item questionnaire that measures alcohol use and related burden in the past year and has shown good psychometric properties to detect early AUD in university students [68, 69] as well as in the general population [70]. The AUDIT identifies hazardous, harmful and dependent drinking patterns, depending on certain cut-of scores. Typically total scores of ≥8 indicates hazardous and harmful alcohol use, although frequently lower and different cut-off scores are suggested, depending on the type of population [68]. We use cut-off scores of ≥5 for women and ≥ 8 for men, based on the Dutch multidisciplinary guideline for AUD [71]. Quality of life will be measured with both the five-level variant of the five-dimensional EuroQol instrument (EQ-5D-5L) and with a Dutch translation of the MOS SF-36 (i.e. RAND-36 item Health Survey) [72].

#### Other variables of interest

Various other variables will be assessed. See Table 2 for an overview of all measurement instruments and assessment timepoints.

- *Socio-demographic information:* General patient characteristics will be collected at baseline, these include: gender, age, marital status, employment status, education level and ethnicity.
- *Drinking motives:* Motives for drinking alcohol will be assessed with the Drinking Motives Questionnaire Revised (DMQ-R) [73]. The DMQ-R is widely used for assessing drinking motives among young populations. The 20-item questionnaire assesses motives for drinking on four factors: enhancing (e.g. drinking because it is fun) coping (e.g. drinking to forget worries), confirmative (e.g. drinking to be liked) and social motives (e.g. drinking to be sociable) [74]. The DMQ-R has shown to be acceptable for measuring drinking motives in adults with mood and anxiety disorders and appropriate for Dutch general adult population [74, 75].
- *General Mental Health:* General mental health is measured with the brief 5-item Mental Health Inventory (MHI-5) [76]. The MHI-5 is a subscale from the MOS SF-36 and has shown to be a good screener for mood disorders in the general population [77].
- *Patient satisfaction:* Participants’ treatment satisfaction is measured with a Dutch version of the 8-item Zufriedenheid (ZUF-8) questionnaire, scores range between 8 and 32, higher scores indicate more satisfaction with the received treatment [78, 79].
- *Intervention uptake:* Intervention uptake is measured through log data from the digital alcohol moderation intervention, for example by the number of times participants logged in, the number of completed modules and program duration.
- *Healthcare utilisation and productivity costs:* Assessment of healthcare costs and productivity losses or gains will be measured with the TIC-P (Trimbos/iMTA questionnaire on Costs associated with Psychiatric illness) a reliable instrument for collecting health care utilization and productivity loss [80].
- *Anxiety symptoms:* Anxiety symptoms will be measured with the brief 7-item Generalized Anxiety Disorder (GAD-7). Total scores range from 0 to 21, higher scores indicate a higher level of anxiety severity, cut-off points of 5, 10 and 15 indicate levels of mild, moderate and severe levels of anxiety respectively [81]. A meta-analysis has found the GAD-7 to be accurate in identifying generalized anxiety disorder and also any anxiety disorder, with high sensitivity and specificity values when using a cut-off point of 8 [82].
- *Traumatic events and post-traumatic stress symptoms:* Traumatic events and post-traumatic stress symptoms are assessed with the Dutch Childhood Trauma Questionnaire – Short Form (CTQ-SF) and Post-traumatic Stress Disorder Checklist for DSM-5 (PCL-5) [83, 84]. The CTQ-SF is a 28-item questionnaire that assesses five dimensions of childhood maltreatment: physical abuse, emotional abuse, sexual abuse, physical neglect and emotional neglect [85]. The Dutch CTQ-SF has been found to have adequate internal consistency and reliability. The 20-item PCL-5 questionnaire is widely used to screen for symptoms of post-traumatic stress disorder in the past month [86]. Several studies have found that the PCL-5 has good psychometric properties [84, 86]
- *Emotion dysregulation:* The widely used Difficulties in Emotion Regulation Scale (DERS-18) will be used to assess difficulties in emotion dysregulation [87]. The DERS-18 is an 18-item short form and has shown good internal consistency and convergent validity in populations with emotional disorders [87].
- *Resilience:* Psychological resilience will be measured with the brief Dutch 9-item Resilience Evaluation Scale (RES). The RES measures two constructs underlying resilience, that is self-confidence and self-efficacy. The instrument is used in various studies and has shown good psychometric properties [88].
- *Borderline personality features:* The 24-item Personality Assessment Inventory-Borderline Features (PAI-BOR) scale will be used to assess borderline features. The PAI-BOR questionnaire screens for borderline personality disorder features [89].
- *Perceived impact of COVID-19 measures:* We used a self-constructed 7-item questionnaire to assess the impact of COVID-19 and related measures on the participants’ depressive symptoms, alcohol use and study participation. The questions assess to what extent the pandemic led to either an increase, decrease or had no impact on depressive symptoms and alcohol consmption and what the reasons for the in-or decrease were. One question assessed whether the pandemic and restricting measures had an influence on any attempts of reducing alcohol use. Lastly, two questions assessed whether the pandemic had any influence on study participation and if so, for which reasons.

**Table 2:** Schedule of enrollment, allocation, interventions, and assessments

	Enrollment	Baseline	Allocation	Follow-up		
	*t*_*-1*_	*t*_*0*_		*t*_*1*_	*t*_*2*_	*t*_*3*_
**Enrollment**						
Eligibility screen	**X**					
Informed consent	**X**					
Baseline assessment		**X**				
Allocation			**X**			
**Interventions**						
TAU + BtB			**X**	**X**	**X**	
TAU			**X**	**X**	**X**	
**Assessments**						
Socio-demographic characteristics		**X**				
TLFB		**X**		**X**	**X**	**X**
AUDIT		**X**		**X**	**X**	**X**
CES-D		**X**		**X**	**X**	**X**
EQ-5D-5L		**X**		**X**	**X**	**X**
RAND-36		**X**		**X**	**X**	**X**
DMQ-R		**X**		**X**	**X**	
MHI-5		**X**		**X**	**X**	**X**
ZUF-8				**X**		
TIC-P		**X**		**X**	**X**	**X**
DERS-18		**X**			**X**	
GAD-7		**X**			**X**	
PAI-BOR		**X**			**X**	
CTQ-SF		**X**				
PCL-5		**X**			**X**	
RES		**X**			**X**	
Impact COVID-19		**X**		**X**	**X**	**X**

*TAU + BtB* Treatment as usual + Beating the Booze, *TAU* Treatment as usual, *TLFB* Timeline Followback, *AUDIT* Alcohol Use Disorder Identification Test, *CES-D* Center for Epidemiological Studies-Depression, *EQ-5D-5L* five-level and five-dimensional EuroQol, *RAND-36* RAND-36 item Health Survey, *DMQ-R* Drinking Motives Questionnaire Revised*, MHI-5* Mental Health Inventory, *ZUF-8* Zufriedenheid questionnaire, *TIC-P* Trimbos/iMTA questionnaire on Costs associated with Psychiatric illness, *DERS-18* Difficulties in Emotion Regulation Scale, *GAD-7* Generalized Anxiety disorder, *PAI-BOR* Personality Assessment Inventory-Borderline Features scale, *CTQ-SF* Childhood Trauma Questionnaire – Short Form, *PCL-5* Post-traumatic stress disorder Checklist for DSM-5, *RES* Resilience Evaluation Scale

### COVID-19 pandemic

Participant recruitment and data collection was partly conducted during the COVID-19 pandemic and the restricting measures, such as periods of social distancing and lockdowns. We have added a COVID-19 questionnaire to the assessments to assess the influence of these restrictions on the primary outcome of the trial. The questionnaire assesses the perceived impact of the pandemic on the participants’ current alcohol use, depressive symptoms and study participation. The external influence of COVID-19 on the primary outcomes will be accounted for in the analysis.

### Data analysis

#### Effectiveness

The primary outcome variable is treatment response, which indicates whether the treatment has been successful or not. We will use Generalized Linear Mixed Models (GLMM) with link functions, depending on the data types and distributions of the dependent variables. For the analyses of the intervention outcomes, we will use count/continuous data in case of alcohol use and dichotomous data in case of treatment response. We used stratified randomization for participating site and we will include the stratas in the GLMM analysis as covariates. Missing data will be handled by using multiple imputation methods. Analyses will be conducted based on intention-to-treat principles and additional sensitivity analyses will be conducted on a protocol/treatment completers sample. All analyses will be carried out using SPSS version 26+ and/or R version 3.0 + .

#### Cost-effectiveness

##### Effects

The treatment response outcome will be used as an effect measure for the cost-effectiveness analysis. For the cost-utility analyses, both the EQ-5D-5L and the RAND-36 health utilities will be used to compute health gains expressed in quality adjusted life years (QALYs).

##### Cost-effectiveness calculations

We will use the Dutch tariffs (utility weights) for the EQ-5D-5L to calculate the QALYs and the Brazier scoring algorithm (SF-6D) in case we report the RAND-36 data [90, 91]. The area under the curve (AUC) method will be used to calculate the weighted utility of the health states over the full 12-month follow-up period and to calculate QALY gains/losses. Likewise, we will calculate cumulative societal costs over the complete follow-up period based on the cost-estimates that were measured at all follow-up assessments. The cost-effectiveness evaluation will be performed and reported in agreement with the CHEERS statement and we will take the societal perspective in addition to a narrower healthcare costs perspective into account [92]. The incremental cost-effectiveness ratio (ICER) will be calculated by dividing the mean difference in costs by the mean difference in effect, that is: ICER = (Costs intervention – Costs control)/ (Effect intervention – Effect control). Confidence intervals will be estimated around the ICER by using a non-parametric bootstrap approach or other robust measures. Differential costs and effects will be plotted on a cost-effectiveness plane. Furthermore, cost-effectiveness acceptability curves will be drawn and one-way sensitivity analyses aimed at assessing the uncertainty around the main cost drivers will be conducted to assess the robustness of the findings.

### Data management

Every included study participant will be given an unique project number for de-identification purposes. The de-identification key will only be accessible for principal investigators, data managers and research assistants of the project. Data collection and management will be conducted with Castor EDC [58]. Data in Castor EDC is stored on servers in the Netherlands and complies with the European General Data Protection Regulation (GDPR) and complies with Good Clinical Practice guidelines. The digital alcohol moderation intervention will be accessible for study participants by logging in on the digital intervention with a personal account. The program and user data from the digital intervention are hosted on a European cloud service provider and complies with the GDPR and other security standards (ISO 27001, NEN 7510). Study outcomes will be anonymously analysed and reported.

## Discussion

Co-occurring depressive disorders and problematic alcohol use are associated with various adverse health outcomes [11–13]. An add-on digital alcohol moderation intervention to depression treatment could be an acceptable and effective treatment for young adults with co-occurring depressive disorders and problematic alcohol use. Currently, availability and evidence on (cost-) effectiveness of such treatment for young adults is insufficient. The current RCT addresses this research gap by evaluating the (cost-) effectiveness of simultaneously adding a digital alcohol moderation intervention to depression treatment (TAU), compared to TAU among young adults with co-occurring depressive disorders and problematic alcohol use. We expect to find a 50% treatment response in the intervention group and a 25% treatment response in the control group. Furthermore, we expect larger improvements in secondary outcome measures and cost-effectiveness in favour of the intervention group.

The current study has several strengths and challenges. Our trial is designed as a pragmatic RCT and aims to evaluate the (cost-)effectiveness of the add-on digital alcohol moderation intervention to treatment as usual for depressive disorders. Pragmatic trials often have higher external validity and generalizability because of conducting the trial in normal clinical practice settings and by using less strict in-and exclusion criteria. Such trial designs therefore often have lower internal validity compared to traditional explanatory RCTs [93]. However, a pragmatic trial design is the most suitable design considering our research aim and the economic evaluation that is conducted alongside the trial. Other strengths of the current study include a multi-centre RCT design, a 12-month follow-up, a relatively large sample size, insight in the (cost-)effectiveness of the combined alcohol and depression treatment for young adults and lastly a novel digital add-on alcohol moderation intervention that was developed through co-creation with expert patients and based on a former and existing web-based alcohol self-help which was found effective among problem drinkers [59]. A common challenge in the field of digital interventions is poor treatment adherence and high dropout rates [94]. Literature shows that guidance can enhance adherence and increase effectiveness compared to completely unguided digital interventions [32, 94]. Thus, minimal asynchronous adherence-focused guidance will be provided by a coach, who will be available for questions and will motivate users to complete the alcohol moderation modules after certain periods of inactivity in the program. Additionally, automatic e-mail reminders will be sent to users after inactivity in the digital intervention. Study drop-out is prevented by giving participants a monetary compensation for every completed follow-up assessment. Completion of follow-up assessments is facilitated by giving participants the opportunity to fill out questionnaires online and on any mobile device. Another challenge for the current study is the COVID-19 pandemic and the corresponding restricting measures that were taken by the Dutch government, such as lockdowns and social distancing. Preliminary research suggest that the COVID-19 pandemic and the restricting measures influence, both negatively and in some circumstances positively, alcohol use and depressive symptoms [95–100]. Therefore, they might also influence the outcomes of the current study. In order to assess the perceived impact of these COVID-19 related measures on both alcohol use and depressive symptoms of study participants, we have added COVID-19-specific items to our online assessments.

If the add-on digital alcohol moderation intervention to TAU for depressive disorders proves to be effective for young adults with co-occurring depressive disorders and problematic alcohol use, dissemination and implementation of the combined treatment in mental health care should be considered. Jellinek, a Dutch health care facility specialized in addiction, is committed to take up the management of the digital alcohol moderation intervention after the research project is finished with acceptable results. This could contribute to sustainable implementation of the digital alcohol moderation intervention in mental health care practice.

## Data Availability

The data that support the findings of this study are available from Arkin but restrictions apply to the availability of these data, which were used under license for the current study, and so are not publicly available. Data are however available from the authors upon reasonable request and with permission of Arkin.

## Abbreviations

AUC: Area Under the Curve
AUD: Alcohol User Disorder
AUDIT: the Alcohol Use Disorder Identification Test
BtB: Beating the Booze
CBT: Cognitive Behavioural Therapy
CES-D: Center for Epidemiological Studies-Depression questionnaire
CTQ-SF: Childhood Trauma Questionnaire – Short Form
DERS-18: Difficulties in Emotion Regulation Scale
DMQ-R: Drinking Motives Questionnaire Revised
EQ-5D-5L: five-level and five-dimensional EuroQol
GAD-7: Generalized Anxiety Disorder questionnaire
GDPR: European General Data Protection Regulation
ICER: Incremental Cost-Effectiveness Ratio
MHI-5: Mental Health Inventory
MI: Motivational Interviewing
PAI-BOR: Personality Assessment Inventory-Borderline Features scale
PCL-5: Post-traumatic Stress Disorder Checklist for DSM-5
QALYs: Quality Adjusted Life Years
RAND-36: RAND-36 item Health Survey
RCT: Randomized Controlled Trial
RES: Resilience Evaluation Scale
TAU: Treatment As Usual
TIC-P: Trimbos/iMTA questionnaire on Costs associated with Psychiatric illness
TLFB: Timeline Followback
ZUF-8: Zufriedenheid questionnaire
